# Complete Genome Sequence of *Streptococcus pyogenes emm28* Clinical Isolate M28PF1, Responsible for a Puerperal Fever

**DOI:** 10.1128/genomeA.00750-15

**Published:** 2015-07-16

**Authors:** Magalie Longo, Mathieu De Jode, Céline Plainvert, Antonin Weckel, Anna Hua, Alice Château, Philippe Glaser, Claire Poyart, Agnès Fouet

**Affiliations:** a INSERM U 1016, Institut Cochin, Unité FRM Barrières et Pathogènes, Paris, France; b CNRS UMR 8104, Paris, France; c Université Paris Descartes, Sorbonne Paris Cité, Paris, France; d Centre National de Référence des Streptocoques, Paris, France; e Hôpitaux Universitaires Paris Centre, Assistance Publique Hôpitaux de Paris, Paris, France; f DHU Risks in Pregnancy, Paris, France; g Institut Pasteur, Unité de Biologie des Bactéries Pathogènes à Gram Positif, Paris, France; h CNRS UMR 3525, Paris, France

## Abstract

We report the sequence of the *Streptococcus pyogenes emm28* strain M28PF1, isolated from a patient with postpartum endometritis. The M28 protein is smaller than that of MGAS6180 (NC_007296.1). Furthermore, the 1,896,976-bp-long chromosome presents, compared to that of MGAS6180, an inversion between the two *comX* genes.

## GENOME ANNOUNCEMENT

*Streptococcus pyogenes* (group A *Streptococcus* [GAS]), an important Gram-positive human pathogen, causes varied clinical manifestations, ranging from noninvasive to invasive diseases and poststreptococcal sequelae, with an estimated 517,000 deaths yearly (1). GAS is genotyped by sequencing the variable regions of the *emm* gene; over 200 GAS *emm* genotypes can be distinguished (2). The most prevalent *emm* types associated with invasive infections in Europe are *emm1*, *emm28*, and *emm89* (3). A tissue tropism and the elicited innate immune response, but not the invasiveness status, are linked to the *emm* type (4, 5), and *emm28* strains display an association with endometritis (3). To study GAS-elicited endometritis, we sequenced the representative GAS strain M28PF1, selected on phenotypic and genotypic bases from a collection of 50 *emm28* independent clinical isolates collected by the Centre National de Référence des Streptocoques (https://cnr-strep.fr) between 2006 and 2009 in France.

Chromosomal DNA was extracted using the MasterPure Gram-positive DNA purification kit (Tebu-Bio) and sequenced using Illumina technology, with read length of 100 nucleotides (nt) and coverage over 200-fold. Libraries were constructed using the Illumina TrueSeq kit. Illumina short reads were assembled using the Velvet software (6). The initial assembly generated 69 contigs of 200 bp to 222 kb. The contigs were ordered by aligning them to the complete genome sequence of strain MGAS6180 (NC_007296.1) using Geneious software (7). Contigs overlapping by more than 13 bp and adjacent in the alignment were joined yielding 21 contigs. The synteny between both chromosomes was tested by performing PCR overlapping all gaps, except that encompassing the two-ribosomal operon locus (nt 16856 to 28476). A PCR product was obtained in all cases but three (see below), and the pairs of amplicons obtained from both strains comigrated, indicating that synteny was conserved around these 17 gaps. The MGAS6180 ribosomal operons and transposase gene sequences were inserted in the corresponding gaps; all other amplified fragments were sequenced.

Overall, in comparison with the MGAS6180 chromosome, there are 30 indels (17 deletions, 13 insertions): 7 in-frame, 7 yielding modified proteins, and 16 intergenic; and 137 single-nucleotide polymorphisms: 40 synonymous, 73 nonsynonymous, and 24 intergenic. The M28PF1 M28 protein lacks one 35 amino-acid repeat.

One failing PCR overlaps the M28_Spy1336 gene encoding the R28 protein, most certainly due to the large number of repetitions. The R28 repetition number varies, yielding proteins of different sizes (8). Western blot analysis carried out on cell-wall extracts from M28PF1 and MGAS6180 strains indicated that the R28 proteins are of the same size (data not shown).

The other two PCRs that failed encompass the two *comX*–ribosomal operon regions which may be chromosomal crossover points (9, 10). PCRs were carried out exchanging the *comX*-proximal primers; DNA fragments were then obtained that comigrated with those produced by the initial primer couples on MGAS6180 DNA, demonstrating that a chromosomal inversion occurred in the M28PF1 genome relative to MGAS6180. This may influence the level of expression of some genes (10).

### Nucleotide sequence accession number. 

This genome sequence has been deposited in GenBank under the accession number CP011535.
